# How Has ‘What Matters to You’ Been Used for Patient Care? A Scoping Review

**DOI:** 10.1111/hex.70217

**Published:** 2025-03-10

**Authors:** Carrie Janerka, Ashley‐Rose Hooper, Belinda Sanders, Olivia Gallagher

**Affiliations:** ^1^ Nursing and Midwifery Research Unit South Metropolitan Health Service Western Australia Australia; ^2^ School of Nursing Curtin University Western Australia Australia; ^3^ Safety Quality and Risk Fiona Stanley Fremantle Hospitals Group Western Australia Australia; ^4^ School of Allied Health University of Western Australia Western Australia Australia

**Keywords:** delivery of healthcare, patient care planning, patient preference, patient‐centred care, review

## Abstract

**Introduction:**

Asking patients ‘What matters to you?’ (WMTY) was first introduced in 2012 as an approach to person‐centred care and has since been integrated into healthcare frameworks internationally. However, it is unclear how extensively and successfully it has been used for patient care. This review aimed to identify and synthesise literature on the use of the WMTY initiative for patient care.

**Methods:**

The scoping review was guided by the Joanna Briggs Institute scoping review methodology. A systematic search of five databases was undertaken using the term ‘What matters to you?’ and limited to articles published between 2012 and 2023 in English. Primary research studies that used WMTY for patient care were included. Article characteristics, as well as interventions and key findings relevant to WMTY, were extracted. Quantitative data were analysed for descriptive statistics (counts and percentages). Qualitative content analysis was used to identify themes relevant to the aim.

**Results:**

Twenty articles were included in the review. WMTY was used for designing person‐centred services, planning individual patient care, and understanding patients and families. Five key themes were identified from findings reported in the articles: (1) What matters to patients?, (2) Benefits of using WMTY, (3) Shortfalls of using WMTY, (4) Facilitators for implementing WMTY and (5) Barriers to implementing WMTY.

**Conclusion:**

WMTY is a simple and versatile tool for supporting person‐centred care, with perceived benefits for consumers, users and organisations. Implementation requires understanding of the phrase and consideration of contextual factors.

**Patient or Public Contribution:**

Consumer representatives were involved in the review of findings, identification of key points for discussion and review of the manuscript.

## 1 Introduction

The notion of asking patients, ‘What matters to you?’ (WMTY) rather than ‘What is the matter with you?’ was first proposed by Barry and Edgeman‐Levitan in 2012 as an approach to shared decision‐making for person‐centred care [1]. The authors suggested that WMTY could support meaningful conversations between healthcare professionals and patients or carers and help identify their individual needs, preferences and goals of care. Talking to the patient and their carers about what matters to them can help plan care that meets their expectations and support their involvement in care. Involving patients in care decisions has many benefits, including enhanced person‐centred communication, reduced patients' concerns about their illness, better satisfaction with care and improved patient and health system outcomes [2, 3]. Working with the patient and seeing them as an individual are central elements for person‐centred care, a widely recognised approach for quality healthcare [4].

The WMTY phrase has been recommended as a person‐centred tool for care conversations and integrated into healthcare frameworks internationally [5]. It has been adopted by health services, institutes and organisations to guide shared decision‐making and care planning with patients across various healthcare settings [6, 7], and for populations with specific care needs, such as older adults and those receiving end‐of‐life care [8]. It has also been promoted as a tool to improve communication between clinicians and patients, enhance patient engagement in care [9], and to better understand and improve clinicians' satisfaction with their work [10]. However, despite global support for the WMTY approach, how it is implemented in practice appears to vary between healthcare organisations.

Recent literature highlights the various ways in which WMTY has been implemented for patient care in healthcare settings. It has been used to identify daily goals for patients in a critical care unit [11], establish priorities for the management of patients undergoing total joint replacement [12] and set long‐term treatment goals for geriatric oncology patients [13]. The WMTY question has also been used to investigate patients' acceptability of prehabilitation for cancer surgery [14] and identify patients' concerns with dialysis care [15]. Healthcare professionals have used WMTY to better understand experiences of therapeutic engagement in mental health wards [16] and to examine what is important for improving transitional care of older patients with chronic illness [17]. The application of WMTY across healthcare contexts appears vast. Systematic exploration of the literature is needed to better understand how WMTY has been implemented for patient care since its inception. To our knowledge, there are no current systematic or scoping reviews on this topic. The aim of this review is to identify and synthesise literature on how the ‘What matters to you?’ initiative has been implemented for patient care.

## 2 Methods

A scoping review approach was used to address the research question as it enables systematic exploration and synthesis of literature to identify available evidence, key ideas about the topic and knowledge gaps [18]. The review was guided by the Joanna Briggs Institute (JBI) scoping review methodology [18] and is reported using the PRISMA‐ScR checklist for scoping reviews [19]. A protocol was registered with the Open Science Framework on January 25, 2024 (https://doi.org/10.17605/OSF.IO/WXZMN).

### 2.1 Eligibility Criteria

Eligibility criteria were defined by [18]: (1) *participants* – patients, carers, healthcare professionals or any other persons who used WMTY for patient care; (2) *concept* – research that involved WMTY or ‘what matters to me?’ for patient care; (3) *context* – hospitals, primary care or any other healthcare setting; and (4) *types of sources* – primary research studies, including experimental and quasi‐experimental study designs, analytical observational studies, descriptive observational study designs, mixed‐method studies, qualitative studies and quality improvement projects. As WMTY was introduced in 2012, articles published from this year were considered [1].

### 2.2 Sources of Evidence

An experienced health librarian developed, in collaboration with the researchers, and executed search strategies in Medline (Table 1), Embase, Emcare, PsycINFO, CINAHL and Web of Science. The search incorporated only the phrases ‘What matters to me?’ and ‘What matters to you?’ across all database fields, prioritising specificity over sensitivity. The searches were limited to publications in English from 2012 to 2023. All searches were executed on the January 12, 2024. Reference lists of relevant articles were hand‐searched for additional sources.

**Table 1:** Medline search strategy.

	Ovid MEDLINE ALL (1946 to January 10, 2024)
1	(‘What matters to you’ or ‘what matters to me’). mp. [mp=title, book title, abstract, original title, name of substance word, subject heading word, floating sub‐heading word, keyword heading word, organism supplementary concept word, protocol supplementary concept word, rare disease supplementary concept word, unique identifier, synonyms, population supplementary concept word, anatomy supplementary concept word]
2	limit 1 to (scalat language and yr = ‘2012 – 2023’)

### 2.3 Evidence Selection

Records identified from database and hand searches were collated, uploaded and managed via EndNote [20], and duplicates were removed. Citations for identified records were exported into an Excel spreadsheet, and their titles and abstracts were screened by two independent reviewers (C.J., A.H.) for assessment against the eligibility criteria. A traffic light system was used to indicate if records were eligible (green), not eligible (red) or unsure (yellow). Uncertainties and any discrepancies were discussed between the reviewers and an agreement was made on records for full‐text retrieval. These articles were read in full and assessed independently against the inclusion criteria by two reviewers (C.J., A.H.), using the same traffic light system. Outcomes were discussed with the research team, and final decisions about inclusion and exclusion were agreed upon by consensus (C.J., A.H., B.S., O.G.).

### 2.4 Data Charting and Synthesis

Data from included articles were extracted by two reviewers (C.J. and A.H.) in duplicate using a data extraction tool created by the research team for the review. The tool captured article characteristics (author, year, title, journal, aim, methods, setting and sample) as well as interventions and key findings relevant to WMTY.

Simple descriptive statistics (counts and percentages) were used to analyse article characteristics and frequency of WMTY use. Qualitative content analysis [21] was used to abstract codes and identify categories and themes for how WMTY was used, and for study findings related to WMTY. Two reviewers (C.J. and A.H.) formulated codes independently then discussed, adapted and agreed on final codes, and repeated this process for the development of provisional categories and themes. Final categories and themes were discussed and agreed upon by the research team (C.J., A.H., B.S. and O.G.) and reviewed by two consumer representatives to ensure clarity and relevance of the data, and to consider meaning of the findings from a patient perspective.

## 3 Results

**Figure 1:**
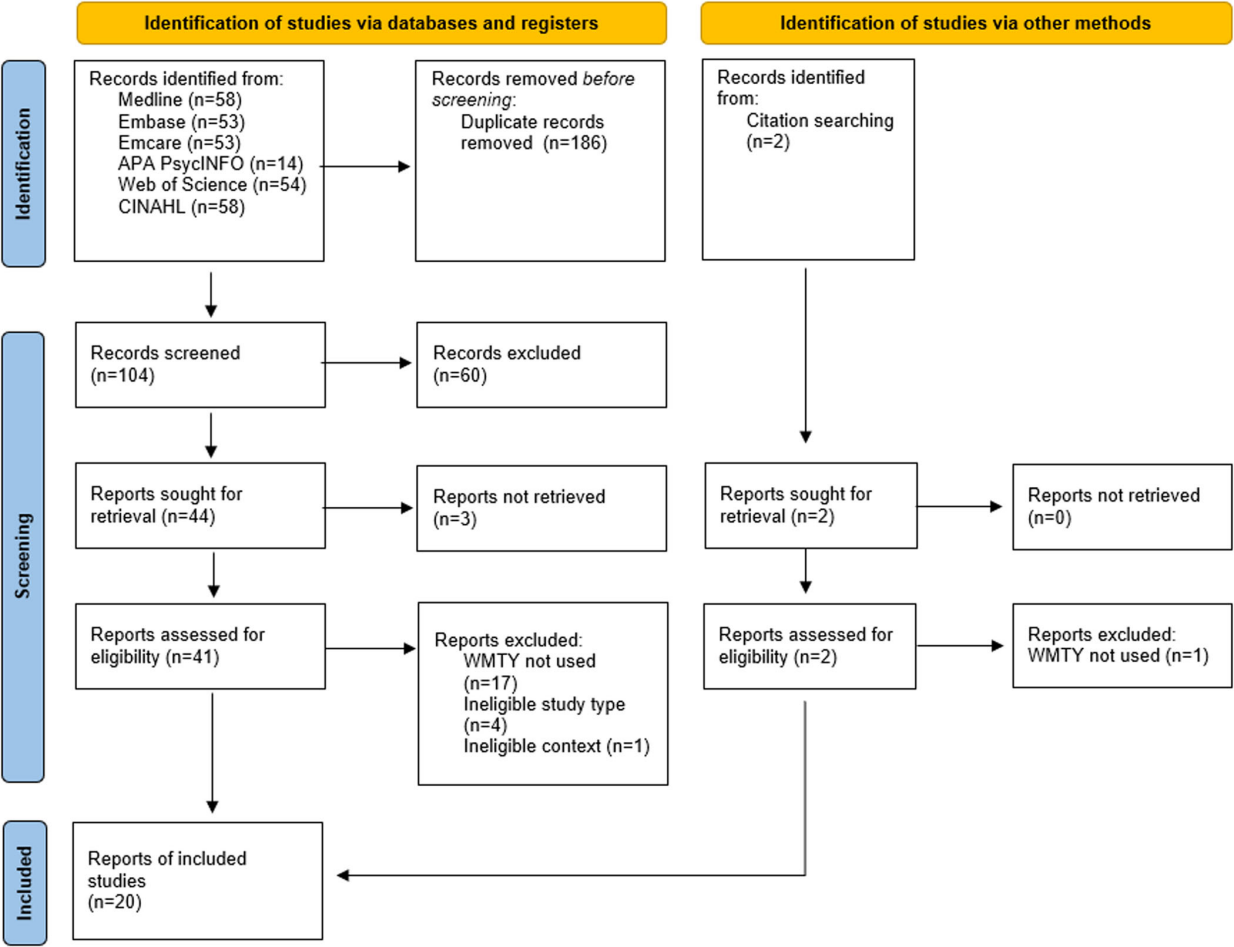
PRISMA flowchart for evidence selection.

Initial searches identified 292 articles, from which 186 duplicates were removed, 60 were excluded during screening of titles and abstracts, 3 were unable to be retrieved and 23 were excluded during full‐text review. Articles were commonly excluded due to ineligible study type (such as a conference abstract) or because the WMTY initiative was not used (the term was cited in the paper title, background, or findings but not used in the study or was coincidental wording). A final 20 articles were included in the review (Figure 1).

### 3.1 Article Characteristics

**Figure 2:**
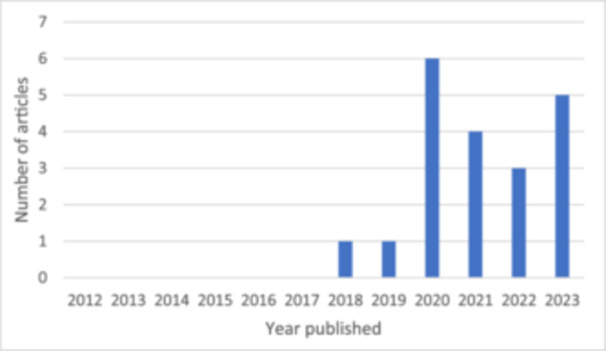
Articles published each year.

Of the included articles, none were published between 2012 and 2017, and most were published in 2020 and 2023 (Figure 2). Articles reported studies conducted in Norway (*n*=10), the United Kingdom (*n* =4), the United States (*n* = 4), Ireland (*n* = 1) and the Middle East (*n* = 1). Study methods were qualitative (*n* = 8), observational (*n* = 4), experimental (*n* = 3), mixed methods (*n* = 2) or quality improvement (*n* = 3). Reported data was qualitative (*n* = 9), quantitative (*n* = 5) or both (*n* = 6). Studies were conducted across a range of healthcare settings: hospital (*n* = 9), home care (*n* = 4), primary care (*n* = 4), clinics (*n* = 3), nursing homes [delivered by health services] (*n* = 3) and community health (*n* = 1) settings. Most study samples involved patients (*n* = 15), and some involved healthcare professionals (*n* = 8), carers, families or friends (*n* = 5), or community members (*n* = 1). Seven studies included a combination of these groups in the sample. Adult patients were the focus of WMTY in the majority of studies (*n* = 16), whilst WMTY for children or adolescent patients were less common (*n* = 4). Patients were often older adults (*n* = 10) and/or patients with chronic or complex needs (*n* = 7). Article characteristics are described in Table 2.

**Table 2:** Article characteristics, WMTY use and study findings.

Author (year)/Country	Aim	Study methods	Setting/Sample	How WMTY was used	Key findings relevant to MWTY
Berntsen et al. [22]/Norway	Improve patient care experience, health outcomes and cost‐benefits for persons with advanced care needs	Quasi‐experimental (control: usual care, intervention: patient‐centred team care)	Hospitals; Primary care services/Patients, older adults with advanced care needs	Part of a patient‐centred model of care that involved goal‐orientated care plans using the WMTY question	Reduction in high‐level emergency care utilisation, increase in low‐level care, and reduced mortality in the intervention group
Berntsen et al. [23]/Norway	Explore the usefulness of a patient‐centred integrated care framework for capturing and guiding care quality	Qualitative (semi‐structured interviews, evaluation of health records)	Hospitals; Primary care services; Community/Patients, older adults with complex needs	Part of a patient‐centred integrated care framework that started with personalised goal setting using the WMTY question	Patient goals were often life related. WMTY may not be straight forward. HCPs generally did not record, share, or evaluate goals
Brand et al. [24]/US	Develop a tool to assess and understand paediatric patients' priorities using WMTM	Observational, descriptive (directed content analysis of text entered in the tool)	Hospital, oncology ward/Patients, paediatric oncology	Bedside poster (visual depiction ofWMTM, 3–5 things important to the patient during their admission)	WMTM themes: (1) identity, (2) communication, (3) care preferences; the tool was feasible, shared top priorities with the care team, and facilitated relationships and engagement in care
Connelly et al. [11]/UK	Understand what mattered to patients daily, personal goals and what patients needed to improve their experience	Quality improvement (thematic analysis of patients' daily goal sheets)	Hospital, critical care unit/Patients, adults with high dependency needs	WMTY asked during daily multi‐disciplinary rounds and recorded on a daily goals sheet	WMTY themes: (1) medical outcomes, (2) the critical care environment, (3) personal care, (4) family and caregivers; WMTY goals enhanced and personalised care and supported humanisation
Corner et al. [25]/Ireland	Evaluate the use and feasibility of implementation of WMTY in orthogeriatric patients	Observational, descriptive (retrospective chart review)	Hospital, acute care/Patients, older adults with hip fractures	WMTY question was part of an orthogeriatric patient assessment proforma	WMTY: return to baseline mobility, getting home, pain relief and family; the tool was feasible and promoted patient‐centred care
Fromme et al. [26]/US	Evaluate acceptability, ease‐of‐use, safety and usefulness of a WMTY book for seriously ill patients	Mixed methods (semi‐structured interviews, web‐based survey)	Community/Patients, adults with serious illness, Carers	8‐page workbook to guide WMTY conversations for advanced care planning	The workbook was an acceptable, easy‐to‐use, safe and useful resources for seriously ill patients and their caregivers
Harding et al. [27]/UK	Tells story of undertaking an ‘always event’ within the radiology department	Quality improvement (codesign of an always event)	Hospital, radiology department/Patients	Asked patients WMTY to identify an always event	Being informed of wait times was important to patients; using WMTY had a positive impact for all involved and led to fewer complaints
Haynes et al. [28]/US	Understand carer, staff and organisation perspectives on the acceptability and feasibility of a WMTY referral system	Mixed methods (retrospective review of health data, interviews, focus groups)	Clinics, women and infants health service/HCPs; Carers (women) of infants	WMTY conversation guide used by nutritionists to identify needs/issues and referral to other services	WMTY was generally acceptable carers, staff and organisations, and initiated referrals to other services; time constraints limited its use
Hirpa et al. [29]/US	Assess patients' priorities for care consultations across five health service delivery domains and potential demographic influence	Observational, descriptive (survey)	Clinic, ambulatory care/Patients, adults	WMTY survey (Likert scale questions with pre‐determined attributes for each health service domain)	WMTY [domains]: humanistic qualities of physicians, leading a healthy lifestyle, shared decision‐making for medications and tests/procedures, and knowledge about insurance coverage; demographics influenced WMTY priorities
Kattouw et al. [30]/Norway	Describe the preferred health service ecosystem for senior citizens living at home from the perspective of stakeholders	Qualitative (interviews, focus groups)	Home care services across a large municipality/HCPs; Community members; Carers	WMTY was an interview question to identify personal goals	WMTY: self‐reliance, remaining active and social, living at home, accessible information and services, continuity of services, compassionate and competent HCPs; WMTY question was perceived as abstract and difficult to answer
Kvael and Olsen [31]/Norway	Explore how patient participation is framed and negotiated within family meetings for intermediate care	Qualitative (observation, analysis against the four habits model)	Nursing home [part of health service], intermediate care/Patients, older adults; Carers; HCPs	WMTY used in family conversations to determine patient's rehabilitation goals	Recommended using WMTY for second habit in the four habits model to elicit patient preferences, views, goals; can be overwhelming if used prematurely in the conversation
Mac Eochagain et al. [13]/UK	Identify patient‐reported treatment goals among new patients, and examine priorities across patient experience domains	Observational, descriptive (survey)	Clinic, oncology/Patients, older adults	WMTY was an open‐ended survey question	WMTY priorities: family/close social network, health and quality of life, functional independence, recreation, psychosocial well‐being, religion/spiritual beliefs, travel and caring for others
Nilsen et al. [32]/Norway	Explore person‐centred care through patients' experiences, participation in care, and WMTY	Qualitative (interviews)	Home care, transitional care (hospital to home)/Patients, older adults	WMTY as the main interview question	WMTY: being seen and respected as a person, informed, and involved in care, and feeling safe in the healthcare system; many participants did not understand the WMTY approach, and some answers were unclear or difficult to translate to goals
Nygaard et al. [33]/Norway	Examine what matters to nursing home residents with dementia regarding healthcare	Qualitative (interviews)	Nursing home/Patients, older adults with dementia	WMTY as the main interview question	WMTY: individuality, personalised care, being seen and heard, empathy, participation in care, have some control/choice regarding everyday life, trusting relationships with HCPs
Oksavik et al. [34]/Norway	Examine how institutional logics materialise in justifications for patient participation within an intervention	Qualitative (case study, document analysis, focus groups, observation)	Hospital; Nursing home; Home care/HCPs; Patients, older adults	Part of (1) a goal‐setting tool for rehabilitation (question during patient meeting), (2) a new patient information form, (3) a care pathway checklist	WMTY promotes shift from medical‐led to person‐centred care; supports skills development; helps focus meetings on patient goals; as checkbox emphasises bureaucracy; in care plan limits goals to being health‐related; mismatch between services available and patient preferences; difficult question to answer
Olsen et al. [17]/Norway	Explore HCPs experiences of using the WMTY question for involving older people in transitional care	Qualitative (observation, interviews)	Home care/HCPs	WMTY question promoted as an approach to patient‐centred care, as part of a national initiative	Successful approach for goal setting, getting to know the patient and building a relationship, complex process that requires competence for use, different interpretations of WMTY, HCP uncertainty role and resources for achieving goals
Pittaway et al. [35]/UK	Design and integrate a tool for ICU patients that helped answer WMTY	Quality improvement (PDSA ‐ develop tool, staff survey)	Hospital, ICU/HCPs; Patients; Carers	Bedside whiteboard with five sections for personal information about the patient	The tool enabled WMTY conversations, helped staff learn about and better care for patients, encouraged compassion, helped reorientate patients, helped engage conversations with patients/families, and was seen as a valuable tool
Schroeder et al. [36]/Middle East	Evaluate the process of using patient‐reported outcomes (PROs) for goal‐directed therapy of hip fracture patients	Quasi‐experimental (sequential interventional controlled trial; control: standard care, intervention: standard care plus WMTY goals)	Hospital, orthopaedic and rehabilitation/Patients, older adults with hip fracture	WMTY question to develop goals and adjust treatment according to goals	WMTY goals: physical function, pain relief, social, emotional, general health, mental health‐related; 39% of patients reached their goals at 4–6 months, 6% not achieved; patients who achieved their goals had better PROs; social and emotional‐mental goals were more often achieved
Tollefson et al. [37]^a^/Norway	Investigate experiences using an idiographic assessment procedure in primary mental health services for adolescents	Qualitative (focus groups, interviews)	Primary care/HCPs	Part of client ‘Assert assessment’: HCPs ask WMTY in first meeting to determine three important issues to address throughout care, issues scored out of 10 each session	Simple question, validated attitudes towards patients, changed HCPs approach, empowered patients, helped identify heart of the issue, helped identify starting point for treatment; formalising WMTY can make it insensitive, conflict between patient needs and HCP responsibilities, sometimes not appropriate, variable use; using a score for meeting WMTY goal showed progress
Tollefson et al. [38]^a^/Norway	Examine the effectiveness of using systematic idiographic assessment to increase adolescents' perceived control of their mental health	Experimental (randomised control trial, control: standard care, intervention: care driven by ‘Assert assessment’ and scores)	Primary care/Patients, adolescents with mental health needs; HCPs	Part of client ‘Assert assessment’: HCPs ask WMTY in first meeting to determine three important issues to address throughout care, issues scored out of 10 each session	Scores for external locus of control were lower in the intervention group (attributing less of their mental health improvement to chance); no significant differences between groups for symptoms of mental health, quality of life or user involvement

Abbreviations: HCP = healthcare professional, ICU = intensive care unit, UK = United Kingdom, US = United States, WMTM = what matters to me, WMTY = what matters to you.

^a^ Related studies.

### 3.2 WMTY Use

The WMTY question was asked by multi‐disciplinary teams (*n* = 6), individual healthcare professionals, such as nurses, doctors, or allied health (*n* = 7), researchers (*n* = 6), self‐reported by patients/clients (*n* = 4) or a mixture of these (*n* = 2). The WMTY question was used to understand people's perspectives and experiences of what mattered to them (*n* = 7) or evaluated as a patient care intervention (*n* = 12). Understanding people's perceptions and experiences involved asking WMTY in an interview (*n* = 3), survey (*n* = 3) or workbook (*n* = 1) to identify what was important to specific patient groups. Interventions involved implementing WMTY in a patient care plan (*n* = 4), communication guide/workbook (*n* = 3), model of care (*n* = 3), bedside poster/whiteboard (*n* = 2), patient assessment form (*n* = 2) and goal‐setting questionnaire (*n* = 1) to guide or improve patient care. One study adopted multiple interventions. The results of one intervention was reported across two studies. Several studies used WMTY to evaluate or inform person‐centred health service design (*n* = 6), and one study identified WMTY in national health guidelines. Interventions were often implemented at a clinical or department level (*n* = 8), whilst some were implemented at an organisational or system level (*n* = 3) or multiple levels (*n* = 1). Themes for how WMTY was used specifically are presented in Table 3.

**Table 3:** How WMTY was used?

Themes	Sub‐themes	Exemplar codes
Designing person‐centred services	To evaluate person‐centred care To support person‐centred service design	To understand person‐centred transitions to home care; to evaluate patient‐centred care
Planning individual patient care	To determine individual patient goals To support care planning To support patient‐practitioner communication	To develop daily goals for patient care; to guide conversations for advanced care planning; to plan patient care
Understanding patients and families	To support patient‐practitioner communication To understand individual patient/family needs	To communicate information about the patient to the HCP; to determine key topics/concerns for the patient

### 3.3 WMTY Findings

Analysis of findings reported in the included articles identified five key themes: (1) What matters to patients?, (2) Benefits of using WMTY, (3) Shortfalls of using WMTY, (4) Facilitators for implementing WMTY and (5) Barriers to implementing WMTY. Themes, sub‐themes, categories and exemplar codes are shown in Table 4.

**Table 4:** Analysis of WMTY findings.

Theme	Sub‐themes	Categories (unique codes)	Exemplar codes
What matters to patients?	Accessing healthcare matters to patients	Accessible healthcare mattered to patients (5)	Accessing services mattered to patients; pain relief mattered to patients
	Person‐centred care matters to patients	Person‐centred care mattered to patients (6) Health and well‐being mattered to patients (4) Information and understanding mattered to patients (3) Involvement in care mattered to patients (3) Life goals mattered to patients (5) Psychosocial needs mattered to patients (6)	Health and well‐being mattered to patients; compassionate and humanistic care mattered to patients; life‐related activities mattered to patients; involvement in their own care mattered to patients
	Therapeutic relationships matter to patients	Therapeutic relationships mattered to patients (2)	Patient–practitioner relationships mattered to patients
Benefits of using WMTY	Perceived consumer benefits	WMTY may improve patient care (5) WMTY supports psychosocial care (1)	WMTY improved/may improve patient outcomes
	Perceived user benefits	WMTY may improve patient care (1)	WMTY supports professional skills of staff
	Perceived organisational benefits	WMTY may improve patient care (2)	WMTY reduced emergency service utilisation
	WMTY facilitating person‐centred care	WMTY supports communication (2) WMTY supports involvement in care (4) WMTY supports person‐centred care (18)	WMTY supported patient empowerment; WMTY supports goal setting/care planning
	WMTY facilitating therapeutic relationships	WMTY supports therapeutic relationships (3)	WMTY helped staff get to know the patient
	Perceived value of WMTY	WMTY is acceptable for patients, families and staff (10)	WMTY is a simple and feasible tool
Shortfalls of using WMTY	Perceived shortfalls of WMTY	WMTY may not improve patient outcomes (1) Conflict between expectations and delivery of WMTY (7)	WMTY goals may not be feasible (beyond scope of the service)
	Challenging to use consistently	Inconsistent use of WMTY by staff (7)	Staff inconsistently recorded WMTY
Facilitators for implementing WMTY	Tailoring WMTY implementation to specific contexts	Adapting WMTY to context as a facilitator (4)	Framing WMTY in the context of services available is helpful
	Integrating WMTY into routine care	Integration of WMTY into care as a facilitator (4)	Integrating WMTY into care framework supported it use
	Strategies to support successful WMTY implementation	Tools for implementing WMTY as a facilitator (5)	Guidance/training on how to use WMTY is helpful
	Understanding the value of WMTY	Understanding and ownership of WMTY as a facilitator (4)	Staff involvement in WMTY design was a facilitator
Barriers to implementing WMTY	Challenging logistics of implementation	Insufficient time and support for WMTY implementation (2)	Poor guidance for using WMTY is a barrier to its use
	Complexity of WMTY model	Complex concept to implement (3) WMTY is complex/not simple to understand (6)	Too much information was a barrier to WMTY

#### 3.3.1 What Matters to Patients?

This theme synthesises findings from studies where patients were asked WMTY. Psychosocial and life‐related needs, such as being at home, spending time with family and friends and being less anxious about their condition, were commonly identified as being important to patients. Maintaining health and well‐being, participating in life activities and being able to access health services were similarly important. Regarding the care they received, patients often desired compassionate and humanistic care, as well as information and being involved in their care. Exemplars of what mattered to patients are presented below.

To be physically able to return to the routine of arranging the house, make flower arrangements and be with family.

(Schroeder et al., [36], p. 5)

Playing tennis and keeping in touch with my friends and of course all my family. Looking after my dog and gardening, but not mowing the lawn!

(Mac Eochagain et al., [13], p. 2)

The cancer nurse was very good in explaining and telling me what I could expect from the Chemotherapy. It made me feel prepared. Now I know that when I feel very bad/frail, it just shows that this thing is working in my body, helping me recover.

(Nilsen et al., [32], p. 574)

It was good to be told my waiting time to be seen. I felt included, treated with respect, kindness and understanding. This makes the process less scary.

(Harding et al., [27], p. S25)

#### 3.3.2 Benefits of Using WMTY

The benefits of using WMTY for patient care were consistently reported across the studies. The initiative was reported or perceived as useful for improving patient care and health outcomes through the evaluation of mortality rates, health service utilisation, patient‐reported outcomes, user experience, observation and WMTY documentation. It supported person‐centred care by facilitating staff communication with the patient; to get to know them as a person and identify their individual goals, priorities and preferences for care. It also supported patient involvement in care and therapeutic relationships, through establishing dialogue, understanding their perspective, and seeing the patient as a whole. The WMTY question was often considered a useful, valuable tool that was acceptable to patients, families and staff. Participants of the included studies shared some of their perceived benefits in the following quotes.

And we would very much like to hear the user's voice and take it into account. And to get to know what matters to the user, we actually have to ask, if not, we are just guessing. And then, it is easy that we guess based on what matters to us instead.

(Olsen et al., [17], p. 9)

So we found that a lot of the referrals led to others […] Other things in people's lives that we were able to assist with, too.

(Haynes et al., [28], p. 7)

It has been a gradual shift, from a national health service with a very paternalistic approach where we know what is best for you. Now it's more like we are more…we are on their team.

(Oksavik et al., [34], p. 10)

It's a very good alternative and it makes it [user involvement] more concrete and tangible, as I said earlier, to the adolescents. Maybe they get a stronger sense of ownership to it in a way. That “these are my sessions, there is that measure again where I have written my topics, it's mine”.

(Tollefson et al., [37]a, p. 7)

#### 3.3.3 Shortfalls of Using WMTY

Issues in using WMTY were highlighted in seven studies. Inconsistent use of the question and poor recording of answers by staff was reported, with medical information sometimes prioritised over WMTY information. Conflict between expectations and delivery of WMTY was reported. Concerns that patient goals may not always be achievable were reported and that standardising use of the question could be perceived as bureaucratic rather than genuine. Using WMTY did not improve patient outcomes in one instance.

Yes, oh yes, but the question is what is important to the patient. If he says ‘It's important I get to rest before I go home,’ should we still listen to the patient's wish, should we work according to the patient's wish or should we work against the patient's wish ‘You have to exercise, you have to go through rehabilitation and make an effort,’ and… it's not easy.

(Oksavik et al., [34], p. 10)

It may be hard for some of the adolescents to answer the question. The ones who are a bit quiet and careful. These are adolescents that find it hard to talk. And for them, I think it's very overwhelming, then you need to help them at least. And you might have to talk a bit about other things as well. It might get too concrete?

(Tollefsen et al., [37], p. 7)

#### 3.3.4 Facilitators for Implementing WMTY

Various facilitators for implementing WMTY were identified. Adapting WMTY to suit the context, involvement of staff in its design and implementation, and the use of resources, training and other tools were important facilitators. Integrating the WMTY question into standard care also supported its use.

It shouldn't just be about, I think, wishing to be able to walk or be able to walk independently and go home, but it should maybe also incorporate existential issues. And I feel kind of in a way that this is something we carry with us from before, but now, it is being opened to encompassing even a bit more.

(Olsen et al., [17], p. 8)

#### 3.3.5 Barriers to Implementing WMTY

Barriers for WMTY use were also noted. Patients and staff reported that WMTY was not straight forward to ask or answer and could be overwhelming or difficult for patients to understand. Staff noted it was complicated to implement in some instances, if WMTY did not translate to tangible care goals. Insufficient time and guidance for using WMTY were also reported barriers.

Now I am speaking for myself, I am afraid to ask for example, “What matters to you?” Yes, what will come out of it? Because it is pretty comprehensive. I have to direct it toward something then. I can ask, “What matters to you?” and think “In relation to what?” In relation to health? In relation to living at home? In relation to what? So, for example, yesterday I thought, “How should I ask that question to someone then? If I do not know myself what I can promise?” If you understand me? If I am to ask someone “What matters to you?” then in a way I have to direct it toward them living longer at home …

(Olsen et al., [17], p. 6)

Many are quick to answer, also when they need home care, what matters to you? Ok, I can answer my son matters to me, my wife matters to me, walking in the mountains matters to me, but our task is not your son, your wife, or the mountains, but what matters? So even here, you have to try to sort things out a bit.

(Olsen et al., [17], p. 7)

No, I don't have any time to talk with the lady about everything, about the mental health, on the communication. But because I'm a student, so sometimes I don't have any time[…].

(Haynes et al., [28], p. 7)

Because we home care nurses don't have the time or space or capacity to treat people differently. We don't care whether you're a king or a hatter. You will get the help you need, what we can provide you with, what you need and what is important to you. (…) There is equal treatment. It doesn't matter who you are. You will get what you need.

(Oksavik et al., [34], p. 10)

## 4 Discussion

This review synthesised literature on how WMTY has been implemented for patient care since the initiative was first introduced in 2012 [1]. Literature indicates recent global adoption, with most articles published since 2020. Application of WMTY was broad and appeared to vary with the aim and context of each study. It was utilised as both a research question and intervention. For research, it helped to better understand the needs of specific patient groups, most commonly older adults, or patients with long‐term care needs. Responses indicated patient needs were multi‐faceted and a desire for a person‐centred approach. As an intervention, WMTY was used as a tool to assist individual care planning or health service improvement. It was implemented at macro levels, such as national guidelines; meso levels, such as models of care and service design; and micro levels, such as care plans and bedside posters. Implementing WMTY had perceived benefits to users, consumers and organisations, particularly for facilitating person‐centred care. And, whilst challenges for using and implementing WMTY were identified, it was often considered a simple, feasible and beneficial tool. Understanding the value of WMTY and contextual strategies for implementation supported its use.

The versatility of the WMTY question is highlighted in this review. Its simplicity allowed it to be used in various contexts and by various health professionals; to facilitate patient‐practitioner communication, plan and evaluate individual patient care, assess care quality, understand patients' needs and design patient‐centred services. The phrase was generally acceptable to patients, family and staff across various countries and suggests mutual understanding and interpretation of the question, though the Norwegian translation ‘what is important to you?’ was noted in some studies [17, 34, 37, 38]. Although described as a simple question, this review also identified the complexities of WMTY. Some studies reported the question was vague or overwhelming, and elicited unrealistic or unfeasible goals for care. Indeed, consumer representatives who reviewed the findings of this review agreed that WMTY could be confusing without sufficient conversation about its purpose and suggested framing the question within the context of care and resources available could facilitate effective use. They considered that each patient is different and may require a tailored approach and level of discussion about what matters to them, including a choice not to answer the question, speaking with family first, and re‐asking the question at different time points. Further, clinicians could liaise with or refer to relevant services if what matters to the patient falls outside of their scope of care.

Despite the few perceived shortfalls, the benefits of using WMTY were considerably more prominent in the articles reviewed. Aligning with Barry and Edgman‐Levitan's conception [1] of WMTY, it was identified as a tool that supported person‐centred care; however, its utilisation extended beyond their initial intent for shared decision‐making. Implementing WMTY also supported patient involvement and empowerment, patient‐practitioner communication and therapeutic relationships, individualised care planning, integration of psychosocial care, and getting to know the patient and their needs. Literature shows that a patient's needs are diverse, and a disease‐focussed approach to healthcare is insufficient at meeting patient needs, particularly for patients with complex needs who may not fit into a standard model of care [39]. Understanding the patient as an individual can help tailor care that is going to be meaningful to the patient and support better quality of care, patient and staff experiences and care efficiencies [4]. This review noted perceived benefits not only to patients (consumers), but also to staff (users) and organisations.

Though there were many perceived benefits of WMTY, comprehensive evaluation of its effectiveness is lacking and an optimal approach for its application is unclear. However, given the broad nature of the concept a ‘one size fits all’ approach would be challenging to apply. It is recognised that regardless of the effectiveness of an intervention, its uptake in practice is largely dependent on contextual barriers and facilitators [40]. This review identified that tailoring WMTY to the setting and resources available, staff involvement, training, tools for implementation and integration of the question into routine care were facilitators; whilst logistical challenges and complexity of the concept were barriers to its implementation. For instance, daily goals, posters and in‐patient care plans lend themselves to acute settings, whilst developing and monitoring long‐term goals may be suited to primary care contexts. Although system‐level support for WMTY was not specifically recognised as a facilitator in the studies reviewed, it is inherent in their publication. Articles were predominantly published in Norway, the United States and the United Kingdom, and align with national initiatives and health reform in these countries preceding this date, particularly involving the care of older people and patients with complex needs [7, 8, 41, 42]. Alas, limiting its application outside of these countries and contexts. Findings of this review suggest the WMTY initiative is one approach to operationalisation of these initiatives.

Finally, the use of WMTY as a research question helped to identify what was important to patients across various contexts. Person‐centred care was a central theme and reflects the multifarious needs of patients, including their physical and psychosocial well‐being, participation in life activities, and understanding of, and involvement in care. Recognising the patient as an individual and working with the patient are key elements of person‐centred care, which can improve outcomes for patients [4]. Findings from this review indicate that using WMTY can support health professionals to work with the patient and align care with the patient's personal goals. Consumer reviewers suggest that personalisation of care could motivate patients on their journey and ‘lift the spirit’. Further, they agreed with the findings that a therapeutic relationship between the patient and practitioner is important and using the WMTY question can help get to know the patient and build this relationship.

### 4.1 Strengths and Limitations

Strengths of this review were the rigorous methodology, including member checking and consensus processes for article selection and data analysis, as well as consumer involvement in understanding the findings. This review also contains some limitations. Despite a systematic search strategy, it is possible that relevant articles were omitted with exclusion of articles not published in English language or if WMTY was not used verbatim. Considering the Norwegian translation of WMTY to ‘what is important to you’, it is possible that studies using only this phrase were missed. Of note was the propensity of interventions and exploration of what matters to older adults and patients with long‐term or complex care needs. Thus, findings of this review are mostly reflective of these populations.

## 5 Conclusion

This review identified WMTY as a simple and versatile tool for identifying patient care goals and supporting person‐centred care. Its application across micro, meso and macro levels of healthcare yielded a range of perceived benefits for the consumer, user and organisation. However, implementation of WMTY requires understanding when and how to use the phrase within each context and for each patient. Operationalisation of WMTY for older adults and people with complex care needs has been the focus of many studies. Further research is needed to understand its use in acute care contexts.

## Author Contributions

**Carrie Janerka:** conceptualisation, investigation, writing – original draft, methodology, project administration, visualisation, data curation, formal analysis. **Ashley‐Rose Hooper:** investigation, validation, formal analysis, writing – review and editing, data curation. **Belinda Sanders:** validation, formal analysis, writing – review and editing. **Olivia Gallagher:** methodology, writing – review and editing, validation, data curation, formal analysis, supervision.

## Conflicts of Interest

The authors declare no conflicts of interest.

## Data Availability

The data that support the findings of this study are available from the corresponding author upon reasonable request.
